# Post-traumatic Anterior Cerebral Artery Rupture after a Severe Traumatic Brain Injury

**DOI:** 10.5005/jp-journals-10071-23114

**Published:** 2019-01

**Authors:** Quentin Mathais, Pierre Esnault, Christophe Joubert, Caroline Dragone, Eric Meaudre

**Affiliations:** 1,2,4 Intensive Care Unit, Sainte Anne Military Hospital, Toulon, France; 3 Departement of Neurosurgery, Sainte Anne Military Hospital, Toulon, France; 5 Intensive Care Unit, Sainte Anne Military Hospital, Toulon, France and French Military Health Service Academy Unit, Ecole du Val-de-Grâce, Paris, France

**Keywords:** Anterior cerebral artery, Blunt cerebrovascular injury, Traumatic brain injury, BCVI: Blunt cerebrovascular injury, TBI: Traumatic brain injury

## Abstract

Blunt cerebrovascular injuries (BCVI) have been increasingly recognized in the past decade due to the initiation of different screening protocols. We present the case of an anterior cerebral artery rupture with free contrast extravasation following a severe traumatic brain injury. Epidemiology, modalities of screening and treatment of BCVI are discussed. This report reminds that the screening of BCVI may be essential after a severe traumatic brain injuries (TBI).

**How to cite this article:**

Mathais Q, Esnault P, Joubert C, Dragone C, Meaudre E. Post-traumatic Anterior Cerebral Artery Rupture after a Severe Traumatic Brain Injury. Indian Journal of Critical Care Medicine, January 2019;23(1):54-55.

Blunt cerebrovascular injuries (BCVI) have been increasingly recognized in the past decade due to the initiation of different screening protocols.^1^ We recently demonstrated that BCVI is frequent after severe TBI, with a prevalence up to 9%.^2^

A 57-year-old woman was admitted to the trauma center for severe TBI after she jumped out of a window. Initial physical examination revealed a Glasgow coma scale score at 3, and her pupils were dilated and nonreactive. She also presented with hemorrhagic shock with diffuse facial bleeding. She was quickly stabilized after transfusion and infusion of norepinephrine. The whole-body computed tomography (CT) scan showed depressed skull fractures, bilateral subdural hemorrhage, intraventricular hemorrhage, diffuse subarachnoid hemorrhage, right frontal contusion, and diffuse brain swelling (Fig. 1). The CT angiography showed a right anterior cerebral artery rupture with blush next to the frontal contusion (Fig. 2). An aneurysm was not present. There was no other vessel injured. There were also multiples facial fractures. Given the severity of the injuries, neurosurgeons did not perform an emergent surgical decompression. Despite advanced medical care, the patient developed refractory intracranial hypertension leading to brain death the fourth day after the admission.

**Figs 1A and B:**
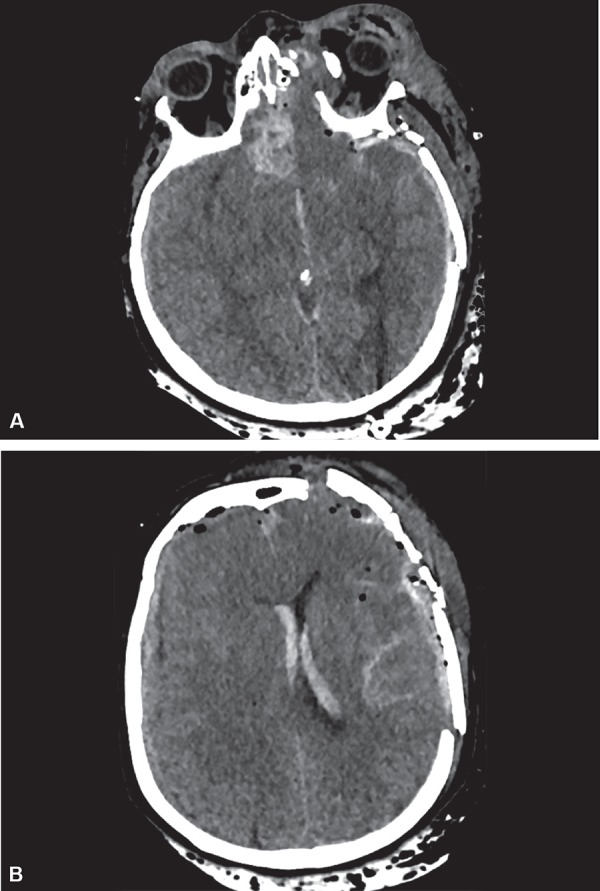
(A) Axial head CT scanner without contrast showing the right frontal lobe contusion; (B) Axial head CT scanner without contrast showing bilateral subdural hemorrhage, intraventricular hemorrhage, diffuse subarachnoid hemorrhage, pneumocephalus associated with depression of the frontal and parietal bones with fracture

Blunt cerebrovascular injuries are not uncommon after blunt trauma. Recent studies found an incidence of approximately 1–2%.^1^ Severe TBI is a well-known risk factor of BCVI, with an incidence up to 5-fold higher in this specific population.^2^ However, catching active bleeding due to an intracranial arterial injury is exceptional. BCVI occurs mainly in large extracranial vessels like carotid and vertebral arteries.^1^ Moreover, most of these cases concern low-grade injuries according to Denver scale. The Denver scale (or Biffl scale) is the most commonly used grading scheme in the literature and has been reused in the 2010 guidelines of the Eastern Association for the Surgery of Trauma to guide treatment (Table 1).^1^ In severe TBI, high-grade injuries (grades IV and V), as in the present report, represent only approximately 20% of the cases.^2^

**Fig. 2:**
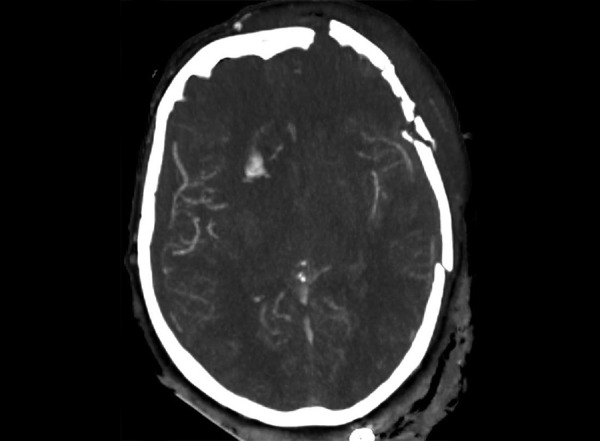
Axial head CT scanner with angiography showing a right anterior cerebral artery rupture with free contrast extravasation

Secondly, this case emphasizes the crucial importance of early detection of BCVI in TBI patients because of its therapeutic impact. Indeed, BCVI is potentially devastating because they can lead to neurovascular events. Untreated BCVIs of the carotid an vertebral arteries could have an overall stroke rate approaching 40 to 60%, and a stroke-related mortality rate as high as 50%.^2,3^ Recent studies showed that medical therapy with anticoagulation or antiplatelet agents that is started before the onset of neurological symptoms could reduce the occurrence of neurological events by a factor of 6, decreasing the stroke rate as low as 4% in grade I to III patients.^4^ Some practitioners could have a doubt concerning the safety of this therapy in patients with severe TBI. However, studies including individuals with neurological injury (TBI or spinal cord injury) did not show an increased risk of intracranial hemorrhage progression after early initiation of pharmacological treatment.^2,3^ In the case of high-grade injuries (grades IV and V), endovascular interventions with coiling could be of interest.

**Table 1:** Denver grading of BCVI

Grade I	Intimal irregularity with <25% narrowing
Grade II	Dissection or intramural hematoma with >25% narrowing
Grade III	Pseudoaneurysm
Grade IV	Occlusion
Grade V	Transection with extravasation

(Source: Bromberg WJ, Collier BC, Diebel LN, Dwyer KM, Holevar MR, Jacobs DG, et al. Blunt cerebrovascular injury practice management guidelines: the Eastern Association for the Surgery of Trauma. J Trauma. 2010;68:587-595)

The modalities of BCVI screening evolved greatly over the past 2 decades. Digital four vessel subtraction angiography has been considered the standard imaging modality.^1^ Nowadays, CT angiography using 32-channel and higher multidetector CT scanners has supplanted subtraction angiography as a first-line screening modality, with a sensitivity of 66–98%, a specificity of 92–100% and a good cost-efficiency ratio.^5^ MR imaging is currently not considered an appropriate tool for BCVI screening. However, it allows a good vessel wall imaging and may be considered for follow up BCVI evaluation.^5^

To our knowledge, this is the first report of a complete anterior cerebral artery transection with free contrast extravasation. This report emphasizes that screening of BCVI may be essential after a severe TBI if there is a suspicion of vascular injury on the initial noncontrast CT scan, and even more with the widespread availability of high-performance CT scanners which permits a fast, efficient and low-cost examination of vascular structures.
